# CTHRC1 targeted by miR-30a-5p regulates cell adhesion, invasion and migration in lung adenocarcinoma

**DOI:** 10.1186/s13019-022-01788-9

**Published:** 2022-03-21

**Authors:** Chaomian Yang, Tianxia Huang, Yue Liang, Yanlong Xue, Ying Liang, Xianqin Wei, Fangchan Meng, Qiu Wei

**Affiliations:** Department of Respiratory Medicine, The First People’s Hospital of Nanning, No. 89 Qixing Road, Nanning, 530022 China

**Keywords:** LUAD, CTHRC1, miR-30a-5p, Invasion and migration, Cell adhesion

## Abstract

The morbidity of lung cancer ranks first among all cancers. Lung adenocarcinoma (LUAD) is a classification of lung cancer, and cell invasion and migration of LUAD are the main causes for its high mortality. Therefore, further exploring the potential mechanism of LUAD metastasis may provide bases for following targeted drug development and treatment of LUAD. In this study, clinical data as well as gene expression profiles were obtained from TCGA-LUAD and GEO to analyze CTHRC1 expression. The result found that CTHRC1 was significantly high in LUAD. Similar results were also discovered in 4 cancer cell lines. Moreover, overexpressed/knock-down CTHRC1 cell lines were constructed. It was uncovered that overexpressing CTHRC1 promoted LUAD cell migration and invasion, and inhibited cell adhesion, while knocked down CTHRC1 had the opposite effect. Afterward, the upstream miRNAs that regulated CTHRC1 were predicted by several bioinformatics websites. It was testified by dual-luciferase method that CTHRC1 was negatively mediated by miR-30a-5p. Overexpressed miR-30a-5p suppressed cell invasion/migration, and increased cell adhesion, while overexpressing CTHRC1 as well reversed such impacts. In conclusion, it was disclosed in this study that CTHRC1 worked as a cancer promoter in LUAD, and miR-30a-5p could target and downregulate CTHRC1 to regulate cell adhesion, and inhibited LUAD cell invasion and migration. These results elucidated at cellular level that upregulated CTHRC1 may be a marker protein for LUAD metastasis.

**Supplementary Information:**

The online version contains supplementary material available at 10.1186/s13019-022-01788-9.

## Introduction

Lung cancer is a recognized human health killer, with its mortality ranks first among all cancers for several years (about 11.6% in 2018) and its morbidity is relatively high [1]. After years of research, lung cancer is divided into many subtypes, one of which is lung adenocarcinoma (LUAD). Over 500,000 people died from lung cancer every year, and the amount has been significantly increasing over the past few decades [2–4]. Although great efforts have been devoted to developing new treatment of lung cancer in recent years, the prognosis of malignant patients remains poor with a 5-year survival rate less than 10% [5, 6]. Shortage of understanding of LUAD-related biological mechanism limits the improvement of therapeutic effect. Hence, it is important to dig related genes of LUAD occurrence and development, and explore its effective mechanism for increasing clinical efficacy.

CTHRC1 is a chondrocyte-secreted glycoprotein first found in the rat balloon-injured artery model and can inhibit collagen matrix synthesis [7, 8]. As revealed in recent years, CTHRC1 is upregulated in various tumors, and promote cancer cell invasion and migration [9–11], which also works as a potential biomarker of various cancers. For example, MEI ZHENG et al. [12] disclosed that CTHRC1 overexpression promotes cervical cancer development by simulating Wnt/PCP signaling pathway. Moreover, upregulated CTHRC1 promotes the invasion of epithelial ovarian cancer via stimulating EGFR signaling pathway. However, CTHRC1 high expression in LUAD might pertain to the angiogenesis of LUAD and indicate poor prognosis of LUAD [13]. But reasons for high expression of CTHRC1 and its regulatory mechanism in LUAD are not clear.

Effects of miRNAs in vivo have been neglected for a long time. However, recent study represented that miRNA mediates gene expression via targeting 3’-untranslated region (UTR) of mRNA [14], so as to modulate the progression of various diseases. Studies revealed that CTHRC1 is regulated by miRNAs in cancers. For example, miR-155 targets CTHRC1 to inhibit colorectal cancer [15]. MiR-30c-mediated CTHRC1 accelerates the metastasis of LUAD cells [16]. MiR-98 targets CTHRC1 to suppress liver cancer cell progression [17]. Nonetheless, there are no reports alike in LUAD. This work scrutinized CTHRC1 and LUAD occurrence and development, and uncovered the molecular mechanism of miRNA targeting CTHRC1, which offers researching directions for targeted treatment of LUAD.

## Materials and methods

### Bioinformatics method

LUAD related data sets were acquired from Gene Expression Omnibus (GEO) and The Cancer Genome Atlas (TCGA), as shown in Table 1. Expression differences of CTHRC1 between normal tissue and LUAD tissue were tested by *t*-test, and the effect of CTHRC1 expression on patient’s prognosis was detected with R “survival” package. Upstream miRNAs that regulated CTHRC1 were predicted with starBase, TargetScan, miRDB and mirDIP databases, and verified via Pearson correlation analysis. Pathway enrichment analysis was undertaken on CTHRC1 by using Gene Set Enrichment Analysis (GSEA) software.

**Table 1:** Sample information related to LUAD of Data Sets from GEO and TCGA databases

Data set	Data type	Normal	Tumor	Follow-up
GSE31210	mRNA	20	226	Yes
GSE32863	mRNA	58	58	No
GSE43458	mRNA	30	80	No
GSE72094	mRNA	0	442	Yes
GSE75037	mRNA	83	83	No
GSE116959	mRNA	11	57	No
GSE119269	miRNA/mRNA	0/0	155/155	No
TCGA-LUAD	miRNA/mRNA	46/59	521/535	Yes

### Cell culture and transfection

LUAD cell lines H1650 (BNCC100260), Calu-3 (BNCC338514), A549 (BNCC337696), H1975 (BNCC100301), and human bronchial epithelial cell line BEAS-2B (BNCC338205) were bought from BeNa Culture Collection. All cells were kept in Roswell Park Memorial Institute (RPMI)-1640 (Thermo Fisher Scientific Company, Waltham, Massachusetts, USA) medium containing 5% fetal bovine serum (FBS), and cultured in an incubator under general conditions.

NC mimic, miR-30a-5p mimic (mimic) were offered by GenePharma (Shanghai, China). oe-CTHRC1 vector, 3 si-CTHRC1 vectors and their negative control lentivirus packing vectors were acquired from Invitrogen (Carlsbad, CA, USA). Vectors were transiently transfected into Calu-3 cells with Lipofectamine 2000 (Thermo Fisher Scientific, Inc.). All cells were cultivated for at least 24 h in the complete medium before transfection, and collected after 36–48 h of transfection.

### qRT-PCR

Total RNA was separated with TRIzol Reagent (Invitrogen). MRNA was reversely transcribed into cDNA with M-MLV Reverse Transcriptase Kit (TaKaRa). MiRNA was reversely transcribed with Superscript II Kit (Invitrogen). PCR system was constructed with miScript SYBR Green PCR Kit (Qiagen, Hilden, Germany). Applied Biosystems 7300 Real-Time PCR System (Applied Biosystems, USA) was applied for qRT-PCR to detect gene expression level, with U6 and GAPDH as the internal references. Relative transcription level of the target gene was calculated with 2^−△△CT^ method. Primer sequences were shown in Table 2.

**Table 2:** Primer sequences

Gene	Primer sequences
CTHRC1	Forward: 5’-TGGACACCCAACTACAAGCA-3’
	Reverse: 5’-GAACAAGTGCCAACCCAGAT-3’
miR-30a-5p	Forward: 5’-GGGCCTGTAAACATCCTCG-3’
	Reverse: 5’-GAATACCTCGGACCCTGC-3’
U6	Forward: 5’-GTGCAGGGTCCGAGGT-3’
	Reverse: 5’-CTCGCTTCGGCAGCACA-3’
GAPDH	Forward: 5’-GGAGCGAGATCCCTCCAAAAT-3’
	Reverse: 5’-GGCTGTTGTCATACTTCTCATGG-3’

### Western blot

After Calu-3 cells were lysed, the protein concentration was measured with bicinchoninic acid (BCA) kit (Thermo, USA). Polyacrylamide gel electrophoresis (PAGE) was applied on 30 μg total proteins, and the proteins were then transferred onto a polyvinylidene fluoride membrane (Amersham, USA). Later, the membrane was blocked with 5% skim milk under room temperature and incubated with primary antibodies at 4 °C overnight after removing the seal solution. The membrane was washed with phosphate-buffered saline with 0.1% Tween-20 (PBST) 3 times, with 10 min of each time. Afterward, the membrane was incubated with horseradish peroxidase labelled secondary antibody for 1 h, and washed with PBST 3 times for 10 min of each time. At last, the membrane was scanned by an optical luminometer (GE, USA) for development. Antibody information was shown in Table 3.

**Table 3:** Antibody information used in western blot

Antibody	Information	Concentration	Company	No
CTHRC1	Polyclonal Rabbit Antibody	1 µg/ml	abcam	ab85739
MMP-2	Polyclonal Rabbit Antibody	1 µg/ml	abcam	ab37150
MMP-9	Polyclonal Rabbit Antibody	1:1000	abcam	ab38898
GAPDH	Polyclonal Rabbit Antibody	1:2500	abcam	ab9485
IgG H&L (HRP)	Goat Anti-Rabbit	1:3000	abcam	ab6721

### Transwell invasion assay

A 24-well Transwell chamber (8 μm aperture, BD Biosciences) was applied here. The upper chamber was coated with Matrigel (Corning, Corning, NY) and the lower chamber was supplemented with DEME medium containing 10% FBS. About 5 × 10^4^ Calu-3 cells were added into the upper chamber. After being cultured at 37 °C for 24 h, cells that did not pass the membrane were removed with a swab applicator. Cells under the membrane were stained with crystal violet (0.3%) and observed under a microscope in 4 random fields to calculate invaded cells.

### Wound healing assay

When Calu-3 cells were grown into about 80% fusion in the well, a 200 μL pipette tip was used to scratch the cell monolayer. The well was washed with medium briefly twice to remove separated cells. Fresh medium was added for another 24 h of cell culture. Cells at 0 h and 24 h were photographed with a microscope for measuring the wound width to calculate cell migratory rate. Migratory rate = (0 h wound width—24 h wound width) /0 h wound width.

### Cell adhesion assay

The 96-well plates were precoated with 100 mg/ml fibronectin at 4 °C overnight and blocked with 1% BSA at 37 °C for 1 h. Next, 2 × 10^4^ Calu-3 cells were inoculated into the 96-well plates and cultured in serum-free DMEM. After 2 h of culture, cells were rinsed with PBS 3 times to gently remove nonadherent cells. Thereafter, attached cells were fixed with 4% paraformaldehyde, and stained with 0.5% crystal violet (Sangon Biotech). The stained crystal violet was dissolved with lauryl sodium sulfate (Amresco, Solon, OH, USA). Absorbance at 570 nm was read with a microplate reader.

### Dual-luciferase reporter assay

Wild-type or mutant 3’UTR sequences of CTHRC1 (CTHRC1 WT, CTHRC1 MUT) were cloned into pmirGLO (Promega, WI, USA) vector to construct 2 luciferase reporter vectors. Taken renilla luciferase expression vector pRL-TK (TaKaRa, Dalian, China) as an internal reference, miR-30a-5p mimic and NC mimic were co-transfected into HEK-293T cells with luciferase reporter vectors, respectively. Dual-luciferase activity detection was conducted based on Dual-Luciferase Reporter Assay System of Promega (Promega, Madison, WI, USA).

### Statistical analysis

All data were treated on SPSS21.0 statistical software (SPSS, Inc, Chicago, IL, USA). Measurement data were displayed as MEAN ± SD and comparison between 2 groups were analyzed by *t*-test. Patient’s overall survival curve was calculated with Kaplan–Meier and patient’s survival differences were analyzed by log-rank. *P* < 0.05 represents that the difference is statistically remarkable, and *p* < 0.01 indicates that the difference is extremely remarkable.

## Results

### CTHRC1 is significantly high and associated with poor prognosis

**Fig. 1:**
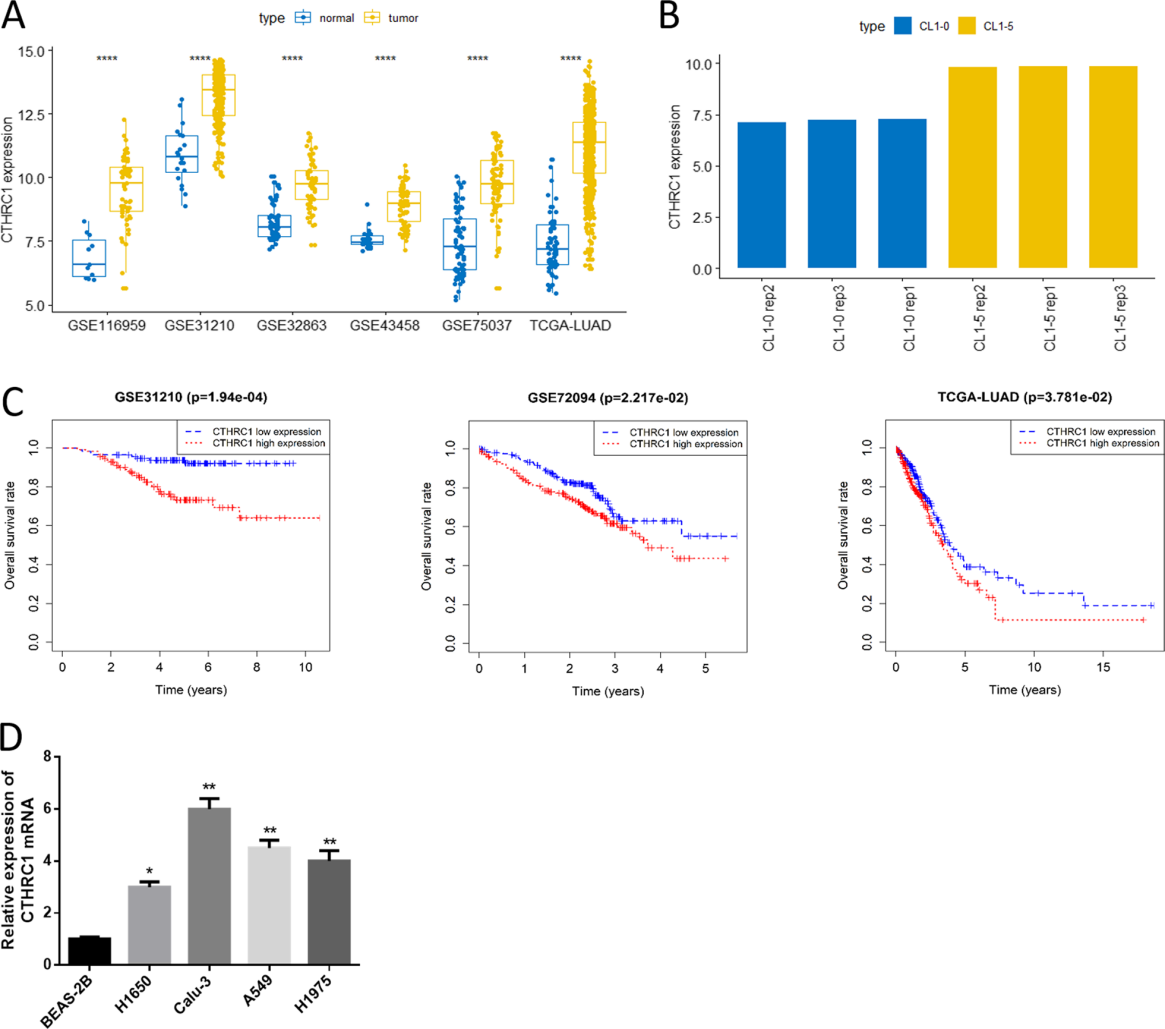
CTHRC1 is significantly high in LUAD cell lines, and related to prognosis of LUAD. **A** Boxplot of CTHRC1 expression in normal and tumor tissues and several GEO data sets (GSE31210, GSE32863, GSE43458, GSE75037, GSE116959); **B** Histogram of CTHRC1 expression in CL1-0 and CL1-5 analyzed by GEO data set GSE42407; **C** Survival curve of CTHRC1 expression on patient’s prognosis in GSE31210, GSE72094 and TCGA-LUAD data sets, with the red line referring to high expression and blue line referring to low expression; **D** CTHRC1 expression in normal human bronchial epithelial cell line and LUAD cell lines; * represents *p* < 0.05, ** represents *p* < 0.01, **** represents *p* < 0.0001

Previous studies indicated that high CTHRC1 expression is closely linked with tumor metastasis [18–21]. However, the action mechanism of CTHRC1 in LUAD is rarely studied, hence it was chosen for research in this study. It was represented by analyzing TCGA-LUAD data set and 3 GEO data sets that CTHRC1 was significantly high in LUAD tissue (Fig. 1A), and CTHRC1 was upregulated in invasive CL1-5 cell line than that in non-migrated CL1-0 cell line (Fig. 1B). Moreover, it was revealed by GEO data sets (GSE31210, GSE72094) and TCGA-LUAD data set with follow-up records that high CTHRC1 expression was remarkably detrimental to patient’s prognosis (Fig. 1C), which indicated that aberrant expression of CTHRC1 may affected LUAD progression. In addition, similar result was found in LUAD cell lines that CTHRC1 expression was higher in 4 LUAD cells than that inBEAS-2B (Fig. 1D). The above results suggested that CTHRC1 was significantly high and related to poor prognosis in LUAD.

### CTHRC1 accelerates LUAD cell invasion and migration, and inhibits cell adhesion

**Fig. 2:**
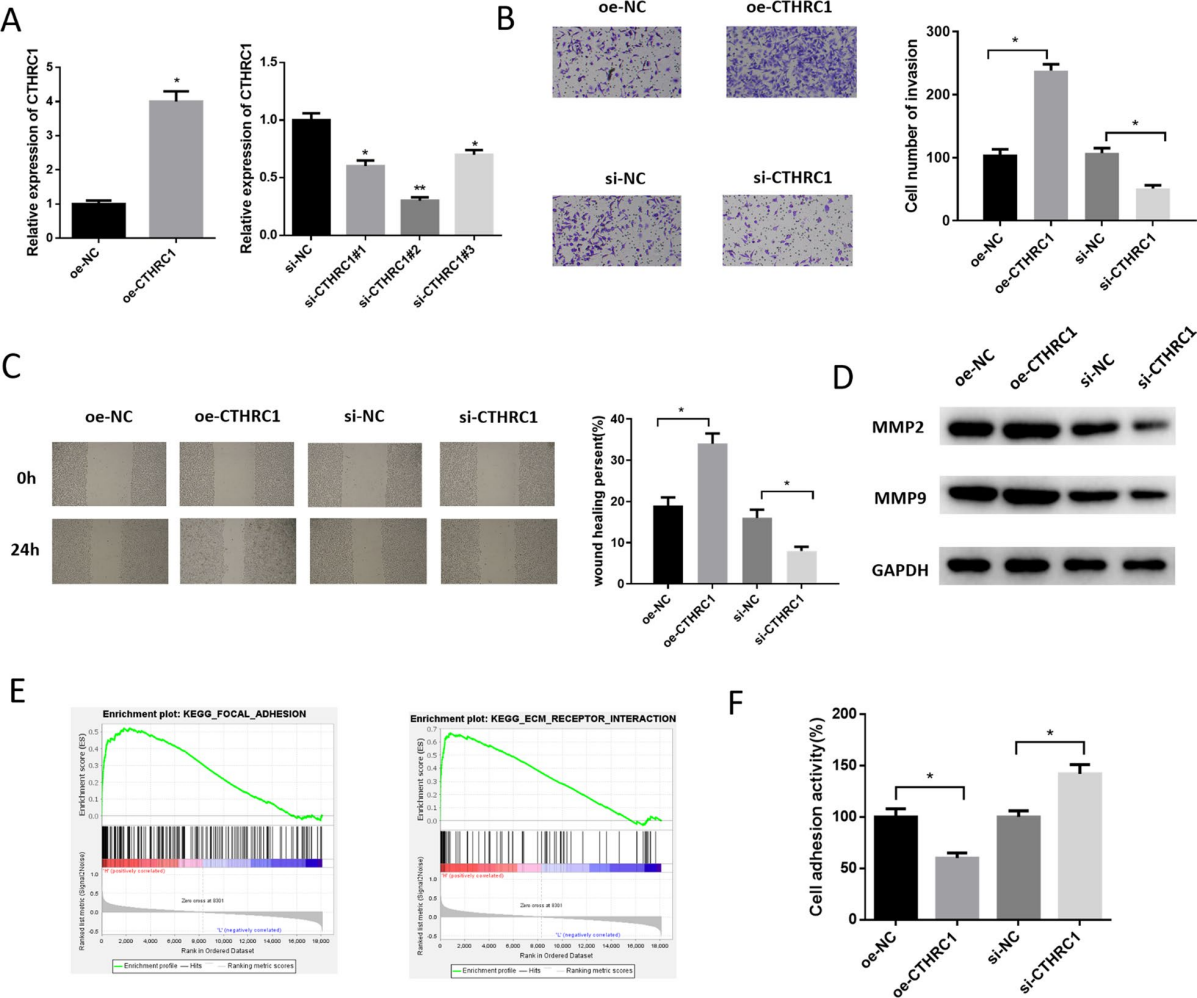
CTHRC1 promotes LUAD cell invasion and migration, and inhibits cell adhesion. **A** CTHRC1 expression in Calu-3 cells in each group; **B**, **C** Changes of invasive and migratory abilities of Calu-3 cells after transfecting overexpressing vector, si-CTHRC1 and its negative control; **D** Expression levels of metastasis-related proteins MMP2 and MMP9 after transfection; **E** Results of GSEA pathway enrichment analysis; **F** The effect of CTHRC1 on cell adhesion detected after transfection; * represents *p* < 0.05, ** represents *p* < 0.01

Studies indicated that high CTHRC1 expression promotes the invasive and migratory abilities of tumor cells [22–24]. To testify that CTHRC1 could regulate LUAD cell invasion/migration, it was overexpressed or knocked down in Calu-3 cell line. Firstly, transfection efficiency was measured. It was found that CTHRC1 expression elevated after transfecting overexpressed vector. The interfering efficiency of si-CTHRC1#2 was the highest, therefore it was chosen for the following experiments (Fig. 2A). Overexpressing CTHRC1 noticeably increased the invasive and migratory capabilities of LUAD cells, while the interference group showed the opposite results (Fig. 2B, C). Hence, it was speculated that CTHRC1 impacted LUAD cell migration and invasion. The expression of tumor metastasis-related proteins supported our speculation. Relative to the control group, the expression of 2 proteins noticeably upregulated in oe-CTHRC1 group while those in si-CTHRC1 group was downregulated (Fig. 2D). Based on above results, it was speculated that CTHRC1 could affect cell invasion and migration in LUAD.

To further study the pathways of CTHRC1 regulating tumor cell invasion and migration, univariate GSEA pathway enrichment analysis was performed. It was suggested that CTHRC1 was remarkably pertinent to extracellular matrix (ECM) receptor interaction and focal adhesion signaling pathways (Fig. 2E). These signaling pathways were closely related to cell adhesion ability, adhesion ability measurement was therefore conducted. Results showed that overexpressed CTHRC1 markedly declined the adhesion ability of LUAD, while interfering CTHRC1 elevated cell adhesion activity (Fig. 2F), which illustrated that CTHRC1 inhibited LUAD cell adhesion.

### CTHRC1 is targeted and modulated by miR-30a-5p

**Fig. 3:**
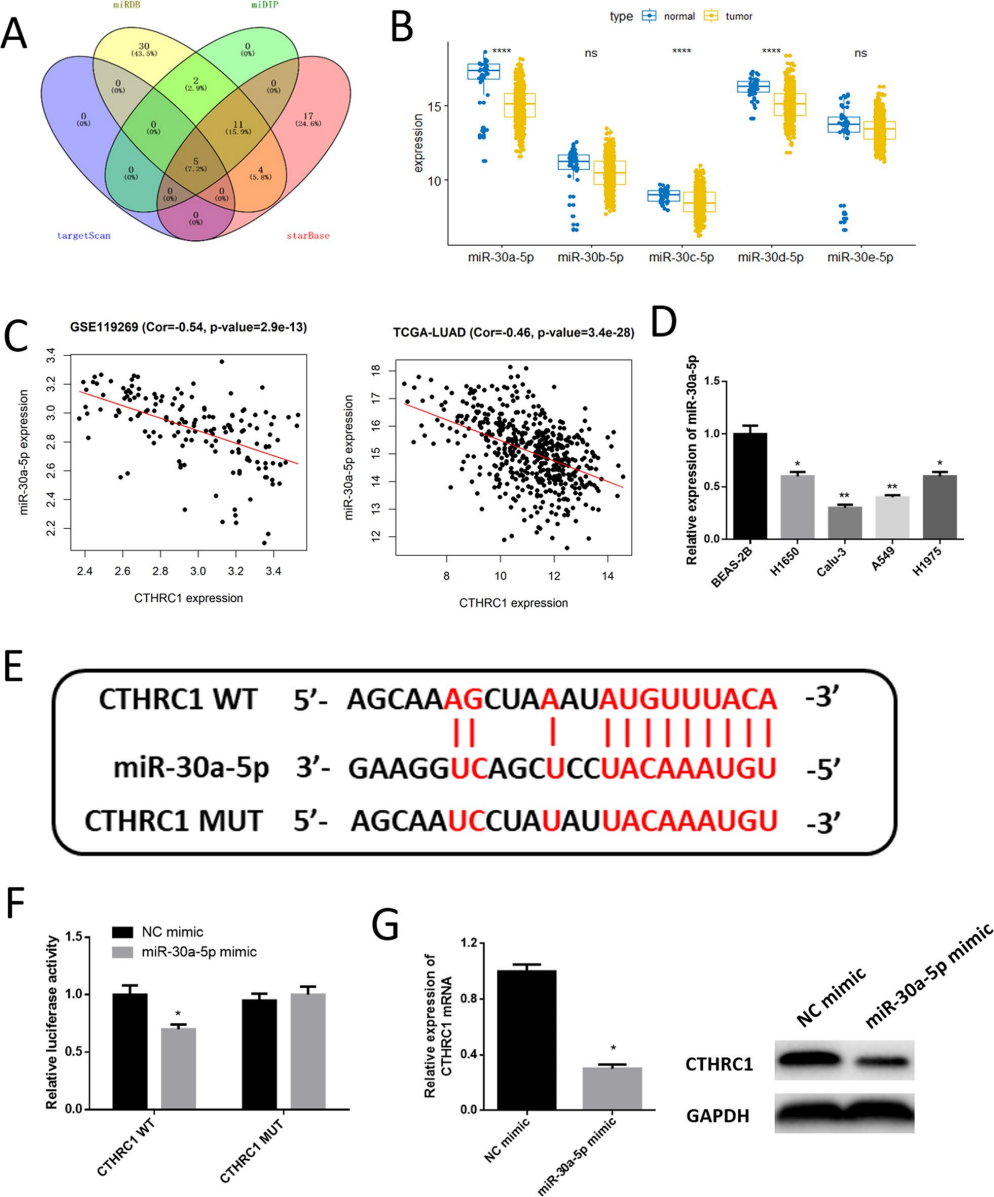
MiR-30a-5p downregulates CTHRC1 expression. **A** Venn plot of target miRNAs predicted via bioinformatics databases; **B** Boxplot of the expression of 5 predicted upstream miRNA in normal tissues and tumor tissues; **C** Pearson correlation analysis between miR-30a-5p and CTHRC1 expression in GSE119269 and TCGA-LUAD data set; **D** The expression of miR-30a-5p in normal human bronchial epithelial cell line and LUAD cell lines; **E** Targeted binding sequences between miR-30a-5p and CTHRC1 predicted with starBase database; **F** Binding relationship between miR-30a-5p and CTHRC1; **G** The expression of CTHRC1 mRNA and protein after overexpressing miR-30a-5p. * represents *p* < 0.05 and ** represents *p* < 0.01

To further research regulatory mechanism of CTHRC1, upstream miRNAs that regulated CTHRC1 were predicted with 4 databases and then intersected to obtain 5 miRNAs (miR-30e-5p, miR-30d-5p, miR-30b-5p, miR-30a-5p, miR-30c-5p,) (Fig. 3A). By detecting mature miRNA data set in TCGA-LUAD, it was manifested that miR-30a-5p was notably low in tumor tissue (Fig. 3B). Fold change |logFC|> 1, while fold change of other miRNAs was less than 1 (Additional file 1: Table S1). The correlation between miR-30a-5p and CTHRC1 was significantly negative (Fig. 3C). Hence, miR-30a-5p was chosen for research. miR-30a-5p low expression was confirmed in cell lines, which was similar to bioinformatics analysis (Fig. 3D). Afterward, binding sequences between miR-30a-5p and CTHRC1 were predicted via TargetScan database (Fig. 3E). Dual-luciferase method validated the reliability of predicted sites. It was represented that the luciferase activity of cells after co-transfecting WT CTHRC1 and miR-30a-5p mimic was markedly decreased, which indicated that miR-30a-5p could targeted CTHRC1 3’UTR (Fig. 3F). Moreover, it was suggested by qRT-PCR and western blot that the expression of CTHRC1 mRNA and protein significantly declined after cells transfecting miR-30a-5p mimic (Fig. 3G). The above results implied that miR-30a-5p was markedly low in LUAD, and could target and downregulate CTHRC1 expression.

### MiR-30a-5p targets CTHRC1 to regulate LUAD cell invasion, migration and cell adhesion

**Fig. 4:**
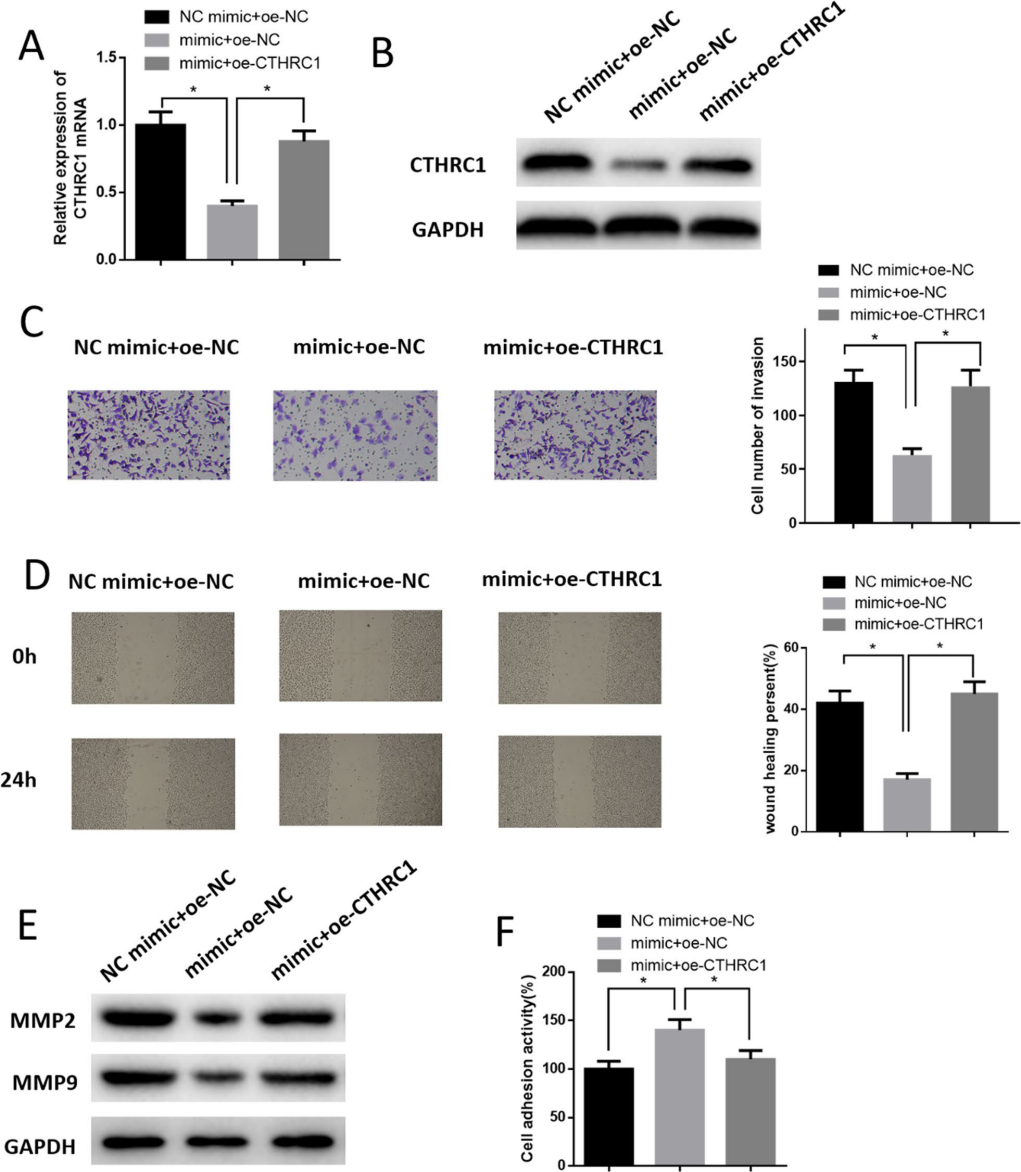
MiR-30a-5p affected the invasion, migration and adhesion of LUAD cells via targeting and downregulating CTHRC1. **A** CTHRC1 expression of Calu-3 cells in each group; **B** CTHRC1 protein expression of Calu-3 cells in each group; **C**, **D** Changes of invasive and migratory abilities of Calu-3 cells in each group; **E** Expression of metastasis-related proteins MMP2 and MMP9 of Calu-3 cells in each group; **F** Cell adhesion of Calu-3 cells in each group. * represents *p* < 0.05

Calu-3 cell lines were divided into 3 groups: mimic + oe-NC group, NC mimic + oe-NC group, and mimic + oe-CTHRC1 group. mRNA and protein levels of CTHRC1 of cells were detected. The result elaborated that overexpressing miR-30a-5p markedly suppressed CTHRC1 expression while simultaneously overexpressing CTHRC1 reversed the results in some degree (Fig. 4A, B). Afterward, invasive and migratory abilities in each group were detected. The result showed that invading cells and migratory rate of cells in mimic + oe-NC group markedly declined (Fig. 4C, D), and MMP2 and MMP9 expression declined (Fig. 4E). However, after simultaneously overexpressing miR-30a-5p and CTHRC1, invasive and migratory abilities of Calu-3 cells were recovered (Fig. 4C, D) and metastasis-related protein expression was also elevated (Fig. 4E). It could be concluded that overexpressing miR-30a-5p strengthened adhesion of Calu-3 cells while overexpressing CTHRC1 reversed that effect (Fig. 4F). Above results proved that miR-30a-5p affected LUAD cell invasion, migration and cell adhesion through targeting and downregulating CTHRC1.

## Discussion

Nowadays, with the development of precise medicine, molecular targeted therapy has been a research hotspot besides conventional therapy. Anaplastic lymphoma kinase (ALK), epidermal growth factor receptor (EGFR), c-ros Repressor of Silencing 1 (ROS1) as crucial lung cancer gene targets have been unanimously recognized by today academic circles, and the era of targeted therapy has begun [24]. More and more biomarkers related to LUAD diagnosis, treatment and prognosis need to be studied. CTHRC1 is an ECM protein associated with atherosclerosis [8], and is upregulated in various cancers and involved in several biological functions of tumors [19, 21, 25, 26], which has value as a potential therapeutic target for cancer. It was manifested by several bioinformatics database profiles and clinical information that CTHRC1 expression was significantly upregulated and related to poor prognosis in LUAD. To further verify the function of CTHRC1, a series of cell experiments in vitro were undertaken. The results indicated that overexpressed CTHRC1 promotes LUAD cell invasion and migration, which coincided with a study about NSCLC [22].

Pathway enrichment analysis was performed for mechanism investigation. It was exhibited that CTHRC1 was mainly enriched in signaling pathways like ECM receptor interaction, focal adhesion, and actin skeleton regulation. These pathways were related to cell adhesion. Proteolytic degradation of the stromal ECM accelerates malignant invasion and metastasis of tumor cells [27, 28]. Additionally, MMPs is a type of zincdependent endopeptidases, and involved in degrading ECM and promoting tumor invasion [29, 30]. After detecting the expression of MMP2 and MMP9 proteins after overexpressing CTHRC1, it was disclosed that the expression of the 2 matrix metalloproteinases was significantly elevated. Moreover, high CTHRC1 expression declined the adhesive ability of LUAD cells, which promoted cancer cell invasion and metastasis.

Our study discovered that CTHRC1 played a vital role in LUAD progression, with the reason for its aberrant expression to be further discussed. MiRNA can target 3’UTR of mRNA so as to regulate mRNA expression. Many studies reported that CTHRC1 is regulated by miRNA in various cancers [15, 16, 31], therefore upstream miRNAs that may target CTHRC1 were predicted via many databases. Only miR-30a-5p was dramatically low in tumor tissues among 5 predicted miRNAs. The binding between miR-30a-5p and CTHRC1 was verified. MiR-30a-5p is found to exert as a cancer-inhibitor in various cancers. For example, CTHRC1 can inhibit tumor growth by suppressing glycolysis via adenosine triphosphate ATP generation, and extracellular acidification rate (ECAR), while increasing oxygen consumption rate OCR in breast cancer cells [32]. MiR-30a-5p inhibits migration of osteosarcoma cells through modulating FOXD1 [33]. In NSCLC, miR-30a-5p suppresses epithelial-mesenchymal transition of cell lines in highly invasive NSCLC via targeting profilin-2 [34], and can strengthen the sensitivity of paclitaxel to NSCLC by targeting BCL-2 expression [35]. In our study, it was uncovered that miR-30a-5p could downregulate CTHRC1 to inhibit LUAD cell invasion and migration, and increase cell adhesive ability. Nevertheless, miR-30a-5p low expression may cause abnormal expression of CTHRC1.

In conclusion, it was revealed in this study that CTHRC1 was remarkably high in LUAD and related to prognosis. High CTHRC1 stimulated LUAD cell invasion and migration, and inhibited cell adhesion. Additionally, CTHRC1 was targeted and negatively regulated by miR-30a-5p to influence LUAD progression. However, the specific regulatory mechanism of CTHRC1 needs to be further researched and it will be further explored in our future studies.

## Supplementary Information

**Additional file 1.** LogFC Value of 5 Predicted Upstream miRNAs That May Regulate CTHRC1.

## Data Availability

The data used to support the findings of this study are included within the article. The data and materials in the current study are available from the corresponding author on reasonable request.
