# Supplementary material for: CTHRC1 targeted by miR-30a-5p regulates cell adhesion, invasion and migration in lung adenocarcinoma
Source: J Cardiothorac Surg. 2022 Mar 21;17:46. doi: 10.1186/s13019-022-01788-9 (PMC8935819; doi:10.1186/s13019-022-01788-9)
Supplement: Supplementary file 1 — Additional file 1. LogFC Value of 5 Predicted Upstream miRNAs That May Regulate CTHRC1. [file 13019_2022_1788_MOESM1_ESM.docx]

**Additional file 1**

**Table S1** LogFC Value of 5 Predicted Upstream miRNAs That May Regulate CTHRC1

| miRNA | logFC |
| --- | --- |
| hsa-miR-30a-5p | -1.73456 |
| hsa-miR-30b-5p | -0.35357 |
| hsa-miR-30c-5p | -0.19833 |
| hsa-miR-30d-5p | -0.8568 |
| hsa-miR-30e-5p | -0.45787 |
